# Stricter Blood Pressure Control Is Associated With Lower Left Ventricular Mass in Children After Kidney Transplantation: A Longitudinal Analysis of the 4C-T Study

**DOI:** 10.1161/HYPERTENSIONAHA.123.21187

**Published:** 2023-07-18

**Authors:** Rizky I. Sugianto, Carl Grabitz, Aysun Bayazit, Ali Duzova, Daniela Thurn-Valsassina, Nima Memaran, Anke Doyon, Nur Canpolat, Ipek Kaplan Bulut, Karolis Azukaitis, Łukasz Obrycki, Ali Anarat, Rainer Büscher, Salim Caliskan, Jérôme Harambat, Francesca Lugani, Zeynep B. Ozcakar, Dušan Paripović, Bruno Ranchin, Uwe Querfeld, Franz Schaefer, Bernhard M.W. Schmidt, Anette Melk

**Affiliations:** Department of Pediatric Kidney, Liver and Metabolic Diseases (R.I.S., C.G., D.T.-V., N.M., A.M.), Hannover Medical School, Germany.; Department of Nephrology and Hypertension (B.M.W.S.), Hannover Medical School, Germany.; Department of Pediatric Nephrology, Faculty of Medicine, Cukurova University, Adana, Turkey (A.B., A.A.).; Division of Pediatric Nephrology, Hacettepe University Faculty of Medicine, Ankara, Turkey (A. Duzova).; Center for Pediatrics and Adolescent Medicine, University Hospital Heidelberg, Germany (A. Doyon, F.S.).; Department of Pediatric Nephrology, Faculty of Medicine, Istanbul University-Cerrahpaşa, Turkey (N.C., S.C.).; Department of Pediatrics, Faculty of Medicine, Ege University, Izmir, Turkey (I.K.B.).; Clinic of Pediatrics, Faculty of Medicine, Vilnius University, Lithuania (K.A.).; Department of Nephrology, Kidney Transplantation and Arterial Hypertension, the Children’s Memorial Health Institute, Warsaw, Poland (L.O.).; University Children’s Hospital, Essen, Germany (R.B.).; Pediatrics Department, Centre Hospitalier Universitaire de Bordeaux, France (J.H.).; IRCCS Istituto Giannina Gaslini, Genoa, Italy (F.L.).; Division of Pediatric Nephrology, Department of Pediatrics, Ankara University Medical School, Turkey (Z.B.O.).; Department of Nephrology, University Children’s Hospital, School of Medicine, University of Belgrade, Serbia (D.P.).; Hôpital Femme Mère Enfant, Hospices Civils de Lyon & Université de Lyon, France (B.R.).; Charité Children’s Hospital, Berlin, Germany (U.Q.).

**Keywords:** cardiovascular disease, hypertension, hypertrophy, left ventricular, pediatric nephrology, prospective study

## Abstract

**BACKGROUND:**

We assessed the effect of blood pressure (BP) control on left ventricular mass index (LVMI) and left ventricular hypertrophy (LVH).

**METHODS:**

Ninety-six patients (64 males) ≥9 months post–kidney transplantation from the 4C-T (Cardiovascular Comorbidity in Children with Chronic Kidney Disease and Transplantation) study were analyzed longitudinally (mean follow-up, 2.6±1.3 years). Cumulative systolic blood pressure (SBP)/diastolic BP exposure was calculated as a time-averaged area under the curve and categorized: ≤50th, 50th to ≤75th, 75th to ≤90th, and >90th percentile (pct). We performed adjusted linear and logistic mixed models for LVMI and LVH, respectively.

**RESULTS:**

At baseline, LVMI was 49.7±12.7g/m^2.16^ with 64% (n=61) kidney transplantation recipients displaying LVH. Compared with patients with cumulative SBP exposure >90th pct, patients with cumulative SBP of 50th to ≤75th showed a significant LVMI reduction of −5.24g/m^2.16^ (*P*=0.007). A similar tendency was seen for cumulative SBP≤50th (β=−3.70 g/m^2.16^; *P*=0.067), but patients with cumulative SBP of 75th to ≤90th pct showed no reduction. A post hoc analysis in patients with cumulative SBP≤75th revealed that median SBP exposure was at 57.5th pct. For cumulative diastolic BP, a significant LVMI reduction was seen in all 3 categories ≤90th pct compared with patients >90th pct. Patients with cumulative SBP of ≤50th or 50th to ≤75th pct showed 79% or 83% lower odds of developing LVH, respectively. Patients with cumulative diastolic BP ≤50th showed a tendency of 82% lower odds for LVH (95% CI, 0.03–1.07).

**CONCLUSIONS:**

Stricter BP control led to regression of LVMI and LVH. Our data suggest a BP target below the 60th pct, which needs to be substantiated in a randomized controlled trial.

**NOVELTY AND RELEVANCE**

**What Is New?**

This study demonstrates the effect of stricter blood pressure (BP) control on left ventricular mass index and left ventricular hypertrophy based on prospectively collected data from pediatric kidney transplantation (KTx) recipients.

Maintaining systolic BP values at ≤75th pct is significantly associated with left ventricular mass index regression and lower odds of developing left ventricular hypertrophy.

A post hoc analysis mirroring the eligibility criteria of a possible randomized controlled trial in pediatric KTx recipients indicates that the protective effect is achieved by maintaining BP values below the 60th pct.

**What Is Relevant?**

Left ventricular hypertrophy is a strong predictor for cardiovascular mortality and affects about 40% of children 1 year after KTx.

BP is the main determinant of left ventricular mass early after KTx.

This analysis offers first evidence supporting lower BP targets in pediatric KTx recipients.

**Clinical/Pathophysiological Implications**

The results of this study underscore the necessity of conducting a randomized controlled trial to validate its findings. This will allow for specific recommendations on BP targets in children and adolescents after KTx.

Life expectancy in children with chronic kidney disease (CKD) after kidney transplantation (KTx) has increased steadily.^1^ Therefore, factors affecting long-term health, especially cardiovascular disease, carry increased relevance as cardiovascular events are among the most common causes of death in pediatric KTx recipients.^2–4^ Cardiovascular morbidity is caused by a variety of cardiovascular risk factors: arterial hypertension is of particular importance and strongly correlated with cardiovascular organ damage.^5,6^ Registry studies demonstrate a high prevalence of arterial hypertension in pediatric KTx recipients^7,8^ with >70% requiring antihypertensive medication^9^ and more than one-third showing actual blood pressure (BP) levels higher than the 95th percentile (pct).^7,8^ We recently extended this finding, demonstrating sharply increased odds for arterial hypertension for patients after pediatric KTx (odds ratio, 88 [95% CI, 44–179]) when compared with healthy age- and sex-matched children from the German Health Interview and Examination Survey for Children and Adolescents in a case-control study.^10^

In children with CKD not yet requiring kidney replacement therapy, the randomized controlled ESCAPE (The Effect of Strict Blood Pressure Control and ACE Inhibition on the Progression of CKD in Pediatric Patients) trial demonstrated that intensified BP control using ACE (angiotensin-converting enzyme) inhibition reduces CKD disease progression.^11^ This led to the implementation of BP goals for patients before kidney replacement therapy. However, currently, no specific recommendations exist on BP management in pediatric patients after KTx that are based on prospective data. This is a major shortcoming as several studies have demonstrated the negative impact of arterial hypertension on cardiovascular health and transplant survival in children after KTx.^12–14^ Left ventricular hypertrophy (LVH), that is, pathologically increased left ventricular mass (LVM), reflects asymptomatic cardiovascular disease^15^ and is the most prominent manifestation of cardiovascular organ damage due to arterial hypertension.^16^ In adults, LVH is associated with death due to major cardiovascular events.^17^ It affects approximately 40% of children 1 year after KTx.^18,19^ We have previously shown that the left ventricular mass index (LVMI) increases in the early course after preemptive KTx and is mainly determined by BP and not by the mode of kidney replacement therapy.^20^

Our main objective was to decipher the range of BP control that can regress or prevent the development of LVH using longitudinal data. We assessed the cumulative exposure of BP on the dynamics of LVMI as well as LVH in a prospective cohort of children from the 4C-T (Cardiovascular Comorbidity in Children with Chronic Kidney Disease and Transplantation) study.^21^ In addition, we simulated the effect of intensified versus regular BP control on LVMI in a subgroup of patients resembling potential inclusion criteria of a randomized controlled trial.

## METHODS

### Data Availability

The data that support the findings of this study are available from the corresponding author upon reasonable request.

### Subjects and Study Design

We included children after KTx from the 4C-T study (which is part of the 4C study^21^) who were transplanted ≥9 months ago and had at least 2 BP and LVMI measurements (Figure S1). After obtaining written informed consent, the 4C study prospectively enrolled 704 patients with CKD (age 6–17 years) with an estimated glomerular filtration rate below 60 mL/min/1.73 m^2^ between 2009 and 2011; 238 received kidney replacement therapy during follow-up. Details on the 4C study set-up, inclusion and exclusion criteria, and data acquisition have been described in detail.^21^ In brief, patients were investigated annually with a full cardiovascular assessment including echocardiography. Seven regional coordinators visited the 55 participating centers in 12 European countries. In addition to annual assessments, blood and urine samples, anthropometric and casual BP measurements, as well as updates of the clinical and medication history were obtained every 6 months. The study fully applies to the Declaration of Helsinki and was approved by institutional review boards of each center as described previously.^21^ The clinical and research activities being reported are consistent with the Principles of the Declaration of Istanbul as outlined in the Declaration of Istanbul on Organ Trafficking and Transplant Tourism.

### LVMI and LVH as Primary and Secondary End Points

Echocardiography was performed following a standardized operating procedure in accordance with the guidelines of the American Society of Echocardiography.^22^ LVM was determined following the modified Devereux formula using the left ventricular end-diastolic dimension, the interventricular septal thickness, and the thickness of the left ventricular posterior wall as follows: LVM (g)=0.80×(1.04 [interventricular septal thickness+left ventricular end-diastolic dimension+left ventricular posterior wall]^3^−[left ventricular end-diastolic dimension]^3^)+0.6.^23^ As our primary end point, LVMI was calculated by dividing LVM in grams by height in meters to the 2.16th with a correction factor of 0.09.^24^ As secondary end point, LVH was defined as an LVMI of ≥45 g/m^2.16^.^24^

### Cumulative Exposure of SBP and DBP

Systolic BP (SBP) and diastolic BP (DBP) were measured using locally available oscillometric devices and defined as the median of 3 measurements.^25^ Table S1 provides the prevalence of hypertension at baseline and the 1st follow-up visit.

#### Calculation

SBP and DBP *z*-scores were calculated, normalized for sex, age, and height.^26^ In patients with an age of >17 years, BP z-scores were calculated using the formula for 17 years of age. The cumulative BP exposure at each LVMI follow-up visit (visit after baseline, vn) was calculated as the cumulative BP area under the curve (AUC) from baseline (v0, first visit at ≥9 months after KTx) to each follow-up visit (vn) averaged by time since v0 to vn (in months).


$$\begin{array}{lll} & Cumulative\;BP\;exposure\;at\;vn\; & \;\;=\frac{\overset{n}{\underset{v=1}{\sum }}AUCn\;\left(z-score.\;months\right)}{time\;from\;v0\;to\;vn\;\left(months\right)} \end{array}$$

For the BP *z*-score AUC, a *z*-score of 0 was set as the baseline for the AUC, that is, the area under 0 was calculated as the area of reduction (as minus). Figure S2 provides further details on the calculation of the cumulative BP exposure.

#### Classification

Cumulative SBP and DBP exposures according to AUC *z*-scores were classified into 4 categories: ≤50th pct, >50th to ≤75th pct, >75th to ≤90th pct, and >90th pct.

### Fixed Covariates

Our main objective was to define the range of cumulative BP exposure that can regress the LVMI or prevent the development of LVH regardless of the potential causes of BP changes, for example, immunosuppressive or antihypertensive treatment. We considered the following parameters as fixed covariates: time since baseline visit, age, sex, estimated glomerular filtration rate,^27^ body mass index *z*-score normalized for age and sex.^28^

### Statistical Analysis

Continuous values are presented as mean±SD, and categories as absolute and relative frequencies. Unpaired *t*-tests were performed to test differences between patients with the presence and absence of LVH at baseline visits. Chi-squared test was used to compare proportions between variables with 2 or more categories. We performed 2 multivariable linear mixed effect models (linear mixed models) for LVMI at follow-up visits (ie, every visit after the baseline visit) to investigate the effect of the cumulative exposure to either SBP or DBP. Models were adjusted for LVMI at baseline and the fixed covariates at the respective follow-up visits. Another 2 multivariable logistic mixed effect models (logistic mixed models) were performed for LVH at follow-up visits to investigate the effect of cumulative exposure to either SBP or DBP. Models were adjusted for LVH at baseline and the fixed covariates at the respective follow-up visits. Due to missing values of fixed covariates, 3 patients were excluded from the mixed model analyses resulting in 215 observations from 93 patients. We included random slopes to account for interindividual variation and time since the baseline visit as a repeated effect to account for intraindividual variation. Unadjusted spline regression was fitted for the LVMI and either cumulative exposure of SBP or DBP. A 2-tailed *P*<0.05 was considered as statistically significant. Statistical analysis was performed using SAS 9.4 (SAS Institute, Cary, NC). This article was written according to the STROBE (Strengthening the Reporting of Observational Studies in Epidemiology) guidelines.^29^

### Sensitivity Analysis

We performed sensitivity analyses for the LVM indexed to the height (m)^2.7^^30^ using similar linear and logistic mixed models for the cumulative exposure of SBP and DBP on LVMI (g/m^2.7^) and LVH, as previously explained in the statistical analysis. In the sensitivity analysis for LVH,^31^ we used the cutoff of >40 g/m^2.7^ in girls and >45 g/m^2.7^ in boys as the definition of LVH.

### Post Hoc Analysis for Trial Simulation

Based on our findings, we then performed a post hoc analysis on a subgroup of patients. This analysis was performed to plan a randomized controlled trial on the effect of intensified versus regular BP control on LVMI. Therefore, the inclusion criteria resemble exactly those of the trial: patients ≤16 years of age who presented with arterial hypertension (defined as SBP or DBP >95th pct or treated with antihypertensive medication) at the baseline visit. Corrected means for LVMI depending on the cumulative SBP category in a total of 94 observations from 37 patients were calculated. Table S2 provides the details on this analysis.

## RESULTS

### Patient Characteristics

Detailed characteristics of the study population are given in Table 1. Of 238 pediatric KTx recipients from the 4C-T study, we included 96 patients (n=67 males) transplanted for ≥9 months, who had at least 2 LVMI and BP measurements (Figure S1). This resulted in a total of 324 observations for this analysis.

**Table 1.**
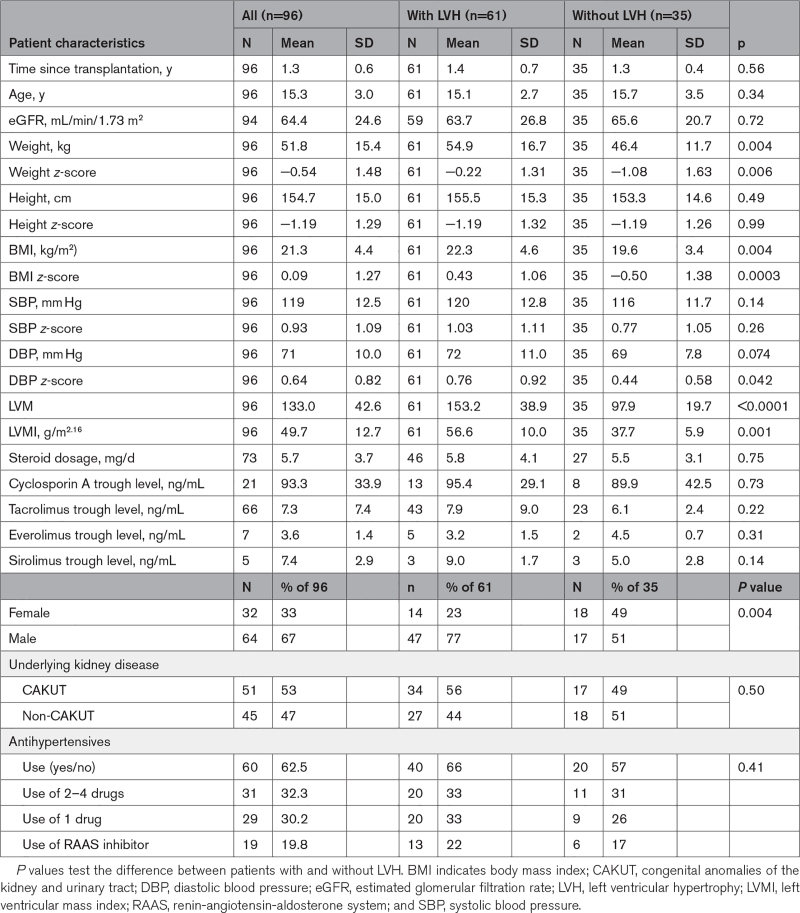
Patient Characteristics for All Children and Differentiated by the Presence or Absence of LVH at Baseline

At baseline, patients were 15.3±3 years old and had been transplanted 1.3±0.6 years ago. Patients were followed for 2.6±1.3 years, with a maximum follow-up of 6.9 years. Twenty-nine patients (30%) were transplanted after prior dialysis. The mean estimated glomerular filtration rate at the baseline visit was 64.4±24.6 mL/min/1.73 m^2^. We identified 61 patients with LVH (77% male), who showed significantly higher body mass index, body mass index *z*-score, and DBP *z*-score compared with those without LVH.

### Cumulative Exposure to SBP and DBP

Figure [A] shows the proportion of patients exposed to the 4 different BP categories differentiated by the presence/absence of LVH for the first follow-up visit. While 43% of patients presenting with LVH had been exposed to SBP >90th pct, this was only the case in 21% of patients without LVH. This difference was even more pronounced for the proportion of patients exposed to DBP >90th pct: this applied to 15% with LVH and only to 2% without LVH.

**Figure.**
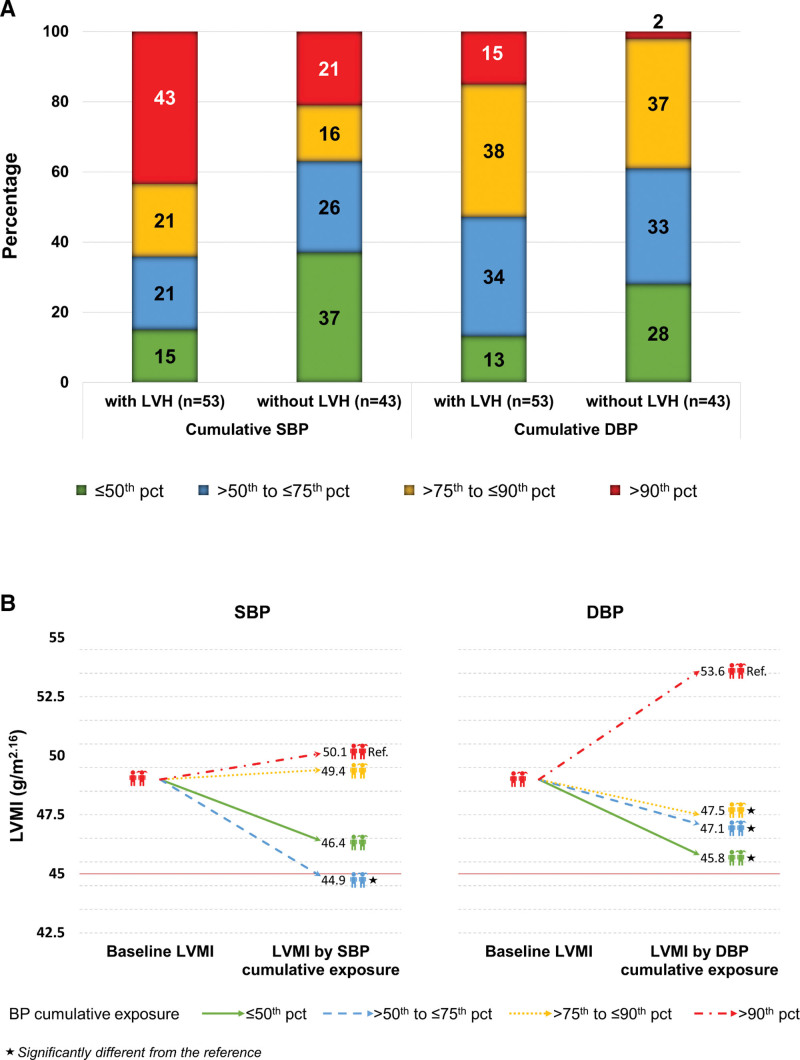
**Blood pressure (BP) exposure and left ventricular structure. A**, Proportion of patients exposed to the 4 different cumulative BP categories differentiated by the presence or absence of left ventricular hypertrophy (LVH). The data presented refers to the first follow-up visit (n=96, 1.1 ± 0.4 years after baseline) and is presented for systolic BP (SBP) and diastolic BP (DBP) separately. B, Simulation of changes in left ventricular mass index (LVMI) according to the categories of either cumulative SBP or DBP exposure. The LVMI at baseline was set as 49 g/m^2.16^. The LVMI after a cumulative exposure of either SBP or DBP at the follow-up visit was calculated based on the respective linear mixed model of LVMI (Tables 2 and 3). The other covariates were held fixed according to mean baseline values: sex=male, time since baseline=2 y, age=15 y, body mass index *z*-score=0, estimated glomerular filtration rate=65 mL/min/1.73 m^2^. The red line at 45 g/m^2.16^ indicates the cutoff value, above which patients are considered to suffer from LVH. pct indicates percentile.

**Table 2.**
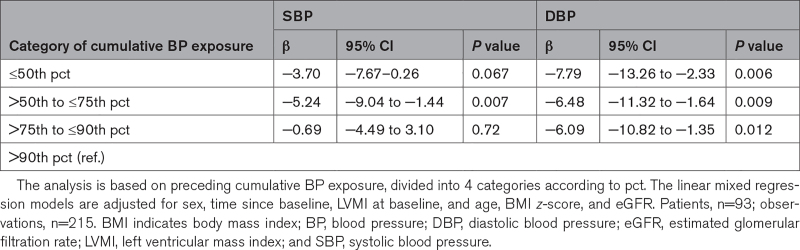
Effect of the Cumulative SBP or DBP Exposure on LVMI

**Table 3.**
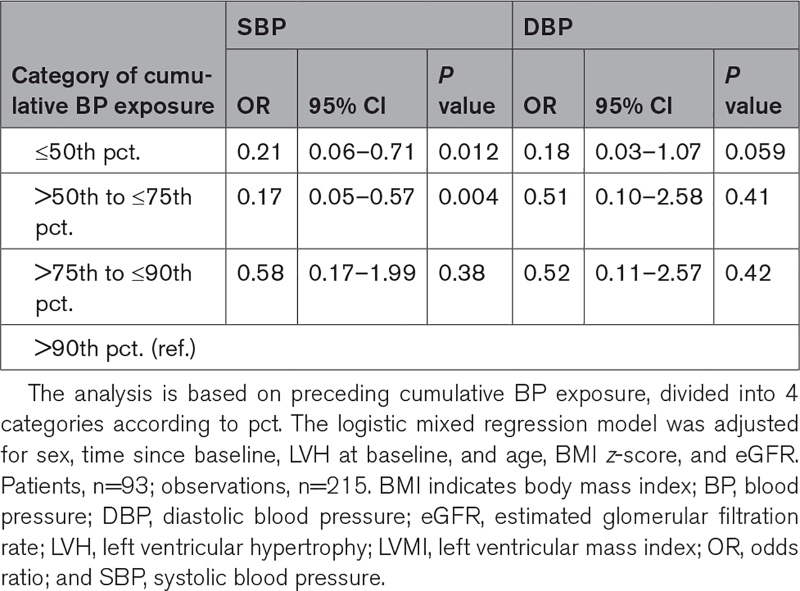
Effect of the Cumulative SBP or DBP Exposure on LVH

### Effect of Cumulative BP Exposure on LVMI

We already saw a differential effect of the cumulative exposure of either SBP or DBP on LVMI in the unadjusted analysis (Figure S3) with an effect for SBP towards an LVMI increase seen already at a lower pct than for DBP. We therefore performed separate analyses for the adjusted models.

Compared with patients with a cumulative SBP exposure >90th pct, patients with cumulative SBP exposure between >50th to ≤75th pct showed a significant LVMI reduction by −5.24 g/m^2.16^ (95% CI, −9.04 to −1.44). A similar tendency was seen in patients with a cumulative exposure below the <50th pct (β=−3.70 g/m^2.16^ [95% CI, −7.67 to 0.26)]. No significant changes were observed in patients who experienced a cumulative SBP between >75th to ≤90th pct (Table 2).

When analyzing the effect of cumulative DBP exposure on LVMI, we found similar magnitudes regarding the LVMI reduction across the categories. Patients with a cumulative DBP exposure of ≤50th pct (β=−7.79 g/m^2.16^ [95% CI, −13.26 to −2.33]), >50th to ≤75th pct (β=−6.48 g/m^2.16^ [95% CI, −11.32 to −1.64]), and >75th to ≤90th pct (β=−6.09 g/m^2.16^ [95% CI, −10.82 to −1.35]) showed a significant reduction when compared with those with a cumulative DBP exposure >90th pct (Table 2).

Figure [B] illustrates these results by showing the longitudinal course of LVMI when a patient is exposed to one of the 4 different categories of cumulative SBP and DBP exposure (with other covariates held fixed).

### Effect of Cumulative BP Exposure on LVH

To estimate the risk of developing LVH, a logistic mixed model was employed. Compared with patients with a cumulative SBP of >90th pct, patients with a cumulative SBP exposure of either ≤50th or >50th to ≤75th pct showed significantly lower odds for developing LVH of 79% (odds ratio, 0.21 [95% CI, 0.06–0.71]) and 83% (odds ratio, 0.17 [95% CI, 0.05–0.57]), respectively. For the cumulative DBP exposure, only those with cumulative DBP of ≤50th pct showed a tendency of an 82% lower odds of LVH (odds ratio, 0.18 [95% CI, 0.03–1.07]; Table 3).

### Sensitivity Analysis

Table S3 provides the results of sensitivity analyses in which we used the LVM indexed for height (m)^2.7^. The results were similar to our main findings showing a cumulative exposure SBP of ≤75th percentile was associated with a lower LVMI and lower odds of developing LVH. The use of LVMI g/m^2.7^ revealed a stronger association of cumulative exposure of DBP with LVMI and LVH.

### Post Hoc Analysis for Trial Simulation

To simulate the conditions for an urgently needed randomized controlled trial on the effect of intensified versus regular BP control, we conducted a post hoc analysis in a subgroup of patients to estimate the achievable LVMI mean values after 2 years (Table S2A). We identified 37 patients (25 males) from the study cohort, who fulfilled the inclusion criteria of our proposed trial at the baseline visit (Table S2B).

We focused on the cumulative SBP exposure as our analyses had shown that SBP was of greater importance for LVMI. When compared with patients with cumulative SBP exposure of >90th pct. (corrected mean, 52.3 [95% CI, 48.7–55.8 g/m^2.16^]) and between >75th and ≤90th pct. (corrected mean 52.7 [95% CI, 46.5–58.8 g/m^2.16^]), those who were exposed to cumulative SBP of ≤75th pct. showed the lowest LVMI values (corrected mean, 47.4 [95% CI, 43.4–51.3]; Table 4). Importantly, a closer look at patients categorized as below ≤75th pct showed a skewed distribution with a median SBP exposure of 57.5th pct (interquartile range: 51^st^ to 66th pct, maximum: 71^st^ pct). Multiple comparisons between the categories in both classifications did not reveal significance highlighting that a larger sample size is needed for an interventional trial.

**Table 4.**
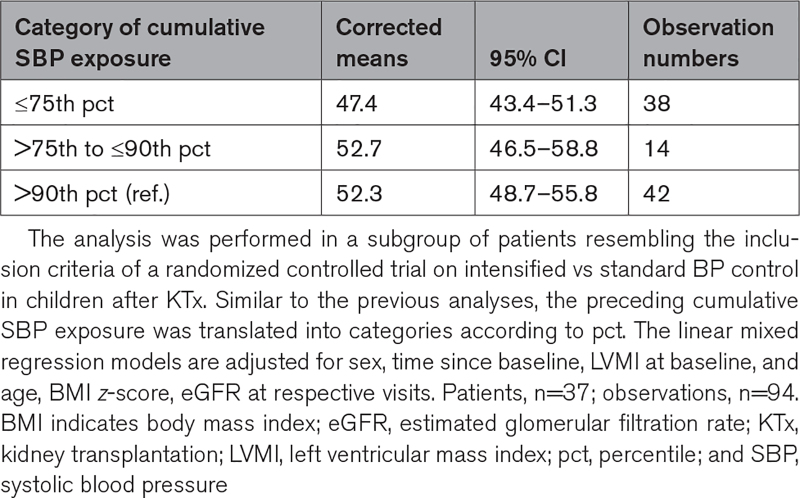
Corrected Means of LVMI Based on Cumulative SBP Exposure in the Trial Simulation Subgroup

## DISCUSSION

This analysis of the prospective observational 4C-T study population intended to explore the magnitude of BP control necessary to prevent the development or trigger the improvement of LVH. We were able to show that a cumulative exposure to SBP values ≤75th pct was associated with a reduction of LVMI, while the SBP exposure between 75th and 90th pct was not associated with lower LVMI. Similarly, patients exposed to SBP ≤50th or between 50th to ≤75th pct had a significant risk reduction of around 80% for the development of LVH. While the cumulative exposure to lower DBP levels was also associated with a lower LVMI, the magnitude of its effect on LVMI across the 3 strata did not largely differ. Our overall findings, therefore, substantiated the influence of continuous exposure to lower SBP levels for the regression of LVM in pediatric KTx recipients. As these data call for a randomized controlled trial exploring the appropriate BP target in this specific population at very high risk, we simulated such an interventional study and were able to support our previous findings that a SBP exposure below the 60th pct is associated with lowest LVMI values.

Mitigating cardiovascular risk in the pediatric KTx population through better BP control is particularly challenging. For the pediatric CKD population an intensified BP control has been shown to delay the progression of kidney disease, a target BP in the low range of normal was associated with a 35% lower risk of progression to end-stage kidney disease within 5 years after initiation.^11^ Regression in LVMI and improvement in myocardial function were shown within 12 to 24 months of good overall BP control under high-dose ACE inhibition in children with CKD.^32^ A small single-center (most likely underpowered) trial including 21 pediatric transplant recipients could not demonstrate an effect of lowering the BP target on graft function.^33^ A large intervention study including 8164 adult participants from the SPRINT (Systolic Blood Pressure Intervention Trial) showed that intensified SBP lowering to <120 mm Hg versus standard SBP of <140 mm Hg reduced the risk of developing LVH by 46% among participants without LVH at baseline, as well as a 66% lower risk among those with the presence of LVH at baseline.^34^ A recent ancillary analysis from the same trial extended these findings: intensified SBP control reduced the incidence of malignant LVH (LVH in combination with elevated cardiac biomarkers reflecting myocardial injury and neurohormonal stress) by 66% and the risk of acute decompensated heart failure by 4.4% in participants with the presence of malignant LVH at baseline.^35^ The HOT-KID (The Hypertension Optimal Treatment in Children with Chronic Kidney Disease) Study, a randomized controlled trial in children with CKD, recently showed a significant reduction of relative wall thickness and a sustained but not significantly different annual reduction of LVMI in children with intensified SBP lowering to <40th pct compared with children with SBP lowering to the 50th to 75th pct. The authors suggest that a target of SBP close to 50th pct optimally regresses LVM.^36^

LVH induced by arterial hypertension is the consequence of pressure overload. Whether SBP or DBP is more important remains a matter of debate. While most studies consider SBP as the underlying force,^34,36,37^ a meta-analysis in adults showed that lowering DBP was more effective than lowering SBP on LVM regression.^38^ We show that both, SBP and DBP reduction, have an effect on LVM, but that a lower SBP than DBP target is necessary to control LVM. Still, the effect of diastolic pressure on the myocardium is detectable. Effects are most likely exerted during end-diastole, when the left ventricular end-diastolic pressure has to increase to open the aortic valve.

Our data mirror the evidence from adult and pediatric trials that a stricter BP control reduces cardiovascular risk. Pediatric KTx recipients are a particularly high-risk group demonstrating cardiovascular morbidity and mortality.^2–4,7,8,10^ Our data strongly suggest that an intensified BP control may reduce cardiovascular organ damage most likely resulting in less long-term morbidity, improved quality of life, and higher life expectancy. Still, we must acknowledge that our results are derived from a prospective observational study thus being subject to potential residual confounding. LVMI might have been influenced by factors, which were not part of our prespecified model building, such as dialysis vintage, anemia, secondary hyperparathyroidism, the immunosuppressive, and antihypertensive therapy. A rather large number of patients remained on steroids in our study. We can assume that BP control might be easier achieved in patients without steroids and on low-dose calcineurin inhibitors because these patients may require fewer antihypertensive drugs.

Certain limitations are inherent to data coming from an observational study. First of all, the data describe an association and cannot infer causality. Longitudinal studies are subject to attrition bias, older participants in our cohort tended to have fewer follow-up visits due to their transition to adult care. The calculated cumulative exposure of BP was based on the standardized office BP measurement. Patients with high BP values only at nighttime would not have been recognized as such and therefore may have diluted the overall positive effect of better BP control on LVMI. Our study has several strengths. All participants were followed longitudinally with standardized cardiovascular measurements. Our pediatric cohort is not confounded by comorbidities related to aging; therefore, the structural changes of the heart are most likely exclusively driven by BP.

Taken together, our findings call for an interventional trial that is designed to define BP targets in children after KTx. The majority of observations in the category ≤75th pct lie below the 60th pct and therefore suggests a BP target ≤60th pct for the intervention group. This would also assure the necessary separation of patients with intensified BP from controls, which most likely will have BP values between the 75th and 90th pct. The type of evidence coming from such a randomized controlled trial would close a major gap of knowledge and address the urgent request made by KDIGO (Kidney Disease Improving Global Outcomes) in the current recommendations.^39^

## CONCLUSIONS

A cumulative exposure to SBP values ≤75th pct was significantly associated with a reduction of LVMI and a risk reduction of around 80% for the development of LVH. Moreover, the post hoc analysis simulating a potential randomized controlled trial confirmed our findings by demonstrating that an SBP exposure below the 60th pct was associated with the lowest LVMI values.

## PERSPECTIVES

Our data call for stricter BP control in pediatric KTx recipients. Ideally, this should be substantiated by a randomized controlled trial addressing the question, which BP target should be achieved in this particularly vulnerable patient group.

## ARTICLE INFORMATION

### Sources of Funding

This study was supported by the German Federal Ministry of Education and Research (No. 01EO0802 to A. Melk); the European Renal Association—European Dialysis and Transplant Association (www.era-edta.org to F. Schaefer); and Roche Organ Transplant Research Foundation (N365520785 to B.M.W. Schmidt). R.I. Sugianto was supported by a fellowship grant from Women in Transplantation (One Lambda, Inc).

### Disclosures

None.

## Supplementary Material
