# Supplementary material for: Stricter Blood Pressure Control Is Associated With Lower Left Ventricular Mass in Children After Kidney Transplantation: A Longitudinal Analysis of the 4C-T Study
Source: Hypertension. 2023 Jul 18;80(9):1900–8. doi: 10.1161/HYPERTENSIONAHA.123.21187 (PMC10424823; doi:10.1161/HYPERTENSIONAHA.123.21187)
Supplement: Supplementary file 1 [file hyp-80-1900-s001.pdf]

Supplementary Materials

Stricter blood pressure control is associated with lower left ventricular mass in children after kidney transplantation: a longitudinal analysis of the 4C-T Study

Rizky Indrameikha Sugianto PhD[1], Carl Grabitz MD[1], Aysun Bayazit MD[2], Ali Duzova MD[3], Daniela Thurn-Valsassina MD[1], Nima Memaran MD[1], Anke Doyon MD[4], Nur Canpolat MD[5], Ipek Kaplan Bulut MD[6], Karolis Azukaitis MD[7], Lukasz Obrycki MD[8], Ali Anarat MD[2], Rainer Büscher MD[9], Salim Caliskan MD[5], Jerome Harambat MD PhD[10], Francesca Lugani MD[11], Zeynep B. Ozcakar MD[12], Dusan Paripovic MD[13], Bruno Ranchin MD[14], Uwe Querfeld MD[15], Franz Schaefer MD[4], Bernhard M. W. Schmidt MD MSc[16*], Anette Melk MD PhD[1*]

1 Department of Pediatric Kidney, Liver and Metabolic Diseases, Hannover Medical School, Hannover, Germany.
2 Department of Pediatric Nephrology, Faculty of Medicine, Cukurova University, Adana, Turkey.
3 Division of Pediatric Nephrology, Hacettepe University Faculty of Medicine, Ankara, Turkey.
4 Center for Pediatrics and Adolescent Medicine, University Hospital Heidelberg, Heidelberg, Germany.
5 Department of Pediatric Nephrology, Faculty of Medicine, Istanbul University-Cerrahpaşa, Istanbul, Turkey.
6 Department of Pediatrics, Faculty of Medicine, Ege University, Izmir, Turkey.
7 Clinic of Pediatrics, Faculty of Medicine, Vilnius University, Vilnius, Lithuania.
8 Department of Nephrology, Kidney Transplantation and Arterial Hypertension, the Children's Memorial Health Institute, Warsaw, Poland.
9 University Children´s Hospital, Essen, Germany.
10 Pediatrics Department, Centre Hospitalier Universitaire de Bordeaux, Bordeaux, France.
11 IRCCS Istituto Giannina Gaslini, Genova, Italy.
12 Division of Pediatric Nephrology, Department of Pediatrics, Ankara University Medical School, Ankara, Turkey.
13 Department of Nephrology, University Children's Hospital, School of Medicine, University of Belgrade, Belgrade, Serbia.
14 Hôpital Femme Mère Enfant, Hospices Civils de Lyon & Université de Lyon, Lyon, France.
15 Charité Children's Hospital, Berlin, Germany.
16 Department of Nephrology and Hypertension, Hannover Medical School, Hannover, Germany.

*equal contribution

Address for correspondence:
Anette Melk, MD PhD
Children’s Hospital, Hannover Medical School
Carl-Neuberg-Str. 1
30625 Hannover
Melk.Anette@mh-hannover.de
Phone: +49-511-532 5597
Fax: +49-511-532 16 5597

Supplemental Table S1. **Prevalence of systolic and diastolic hypertension. Data is presented as frequency (%) for baseline and 1st follow-up (FU) visits.**

Blood pressure categories	Systolic BP		Diastolic BP	
	Baseline	1st FU	Baseline	1st FU
**Normotension**<br>BP<95th pct	30	31	36.5	32.3
**Controlled hypertension**<br>BP<95th pct with antihypertensive therapy	40	46	51	54.2
**Uncontrolled hypertension**<br>BP>95th pct with antihypertensive therapy	23	17	11.5	8.3
**Untreated hypertension**<br>BP>95th pct without antihypertensive therapy	7	6	1	5.2

Blood pressure is categorized based on values according to pct and the use of antihypertensive therapy.

Abbreviations: BMI, body mass index; DBP, diastolic blood pressure; eGFR, estimated glomerular filtration rate; LVMI, left ventricular mass index; pct, percentile; SBP, systolic blood pressure.

Supplemental Table S2. **Details on post-hoc analysis for trial simulation and patient characteristics of the trial simulation sub-group at baseline.**

A. Post-hoc analysis for trial simulation

**Inclusion**	**We included 37 patients with an age ≤16 years, who presented with arterial hypertension (defined as SBP or DBP >95th pct. or treated with antihypertensive medication) at the baseline visit.**
**Exposure**	We focused on cumulative SBP exposure and used 3 categories that closely resembled our main analysis for LVMI with the exception that we combined the categories ≤50th pct and >50th-≤75th pct into one category.<br><br>The following categories were used:<br>"≤75th pct",<br>">75th to ≤90th pct",<br>">90th pct".
**Endpoint**	LVMI
**Analysis**	The aim of this analysis was to estimate the LVMI after a certain cumulative SBP exposure (as categorized above). We calculated the corrected means of LVMI for each category of the cumulative SBP exposure based on a linear mixed model. The model was adjusted for LVMI at baseline, time since baseline, age, sex, eGFR, and BMI z-score at the respective follow-up visits.

B. Patient characteristics of the sub-group (N=37) used for trial simulation at baseline

**Patient characteristics**	**Mean**	**SD**
Time since transplantation (years)	1.3	0.8
Follow-up time (years)	2.9	1.3
Age (years)	13.7	2
eGFR (mL/min/1.73m^2)	64.7	17.4
BMI (kg/m2)	21.2	4.63
BMI z-score	0.34	1.17
SBP (mmHg)	121	11
SBP z-score	1.32	1.05
DBP (mmHg)	70	11
DBP z-score	0.64	0.91
LVMI (g/m$^{2.16}$)	51.8	13.6

Abbreviations: *BMI, body mass index; DBP, diastolic blood pressure; eGFR, estimated glomerular filtration rate; LVMI, left ventricular mass index; pct, percentile; SBP, systolic blood pressure.*

Supplemental Table S3. Sensitivity analysis showing the effect of the cumulative systolic or diastolic BP exposure on LVMI g/m$^{2.7}$ and LVH.

A. Effect of cumulative BP exposure on LVMI g/m$^{2.7}$

Category of cumulative BP exposure	SBP			DBP		
	β	95% CI	p	β	95% CI	p
≤50th pct	-2.73	-6.05 – 0.58	0.100	-6.14	-10.69 – (-1.60)	0.009
>50th to ≤75th pct	-3.85	-7.00 – (-0.69)	0.017	-4.86	-8.89 – (-0.83)	0.019
>75th to ≤90th pct	-0.40	-3.57 – 2.77	0.800	-4.76	-8.71 – (-0.82)	0.018
>90th pct (ref.)	0			0		

The analysis is based on preceding cumulative BP exposure, divided into 4 categories according to pct. The linear mixed regression models are adjusted for sex, time since baseline, LVMI at baseline, and age, BMI z-score, and eGFR.
Patients n=93, observations n=215.

B. Effect of cumulative BP exposure on LVH.

The presence of LVH is defined as LVMI >40 g/m$^{2.7}$ in girls and >45 g/m$^{2.7}$ in boys.

Category of cumulative BP exposure	SBP			DBP		
	OR	95% CI	p	OR	95% CI	p
≤50th pct	0.28	0.08 – 0.98	0.047	0.08	0.01 – 0.45	0.005
>50th to ≤75th pct	0.28	0.09 – 0.91	0.035	0.13	0.03 – 0.59	0.008
>75th to ≤90th pct	1.19	0.38 – 3.71	0.760	0.22	0.05 – 0.95	0.043
>90th pct (ref.)	0			0		

The analysis is based on preceding cumulative BP exposure, divided into 4 categories according to pct. The logistic mixed regression model was adjusted for sex, time since baseline, LVH at baseline, and age, BMI z-score, and eGFR.
Patients n=93, observations n=215.

Abbreviations: β, regression coefficient; BMI, body mass index; BP, blood pressure; CI, confidence interval; DBP; diastolic blood pressure; eGFR, estimated glomerular filtration rate; LVH, left ventricular hypertrophy; LVMI, left ventricular mass index; OR, odds ratio; SBP, systolic blood pressure

Supplemental Figure S1. Flow chart depicting the study population.

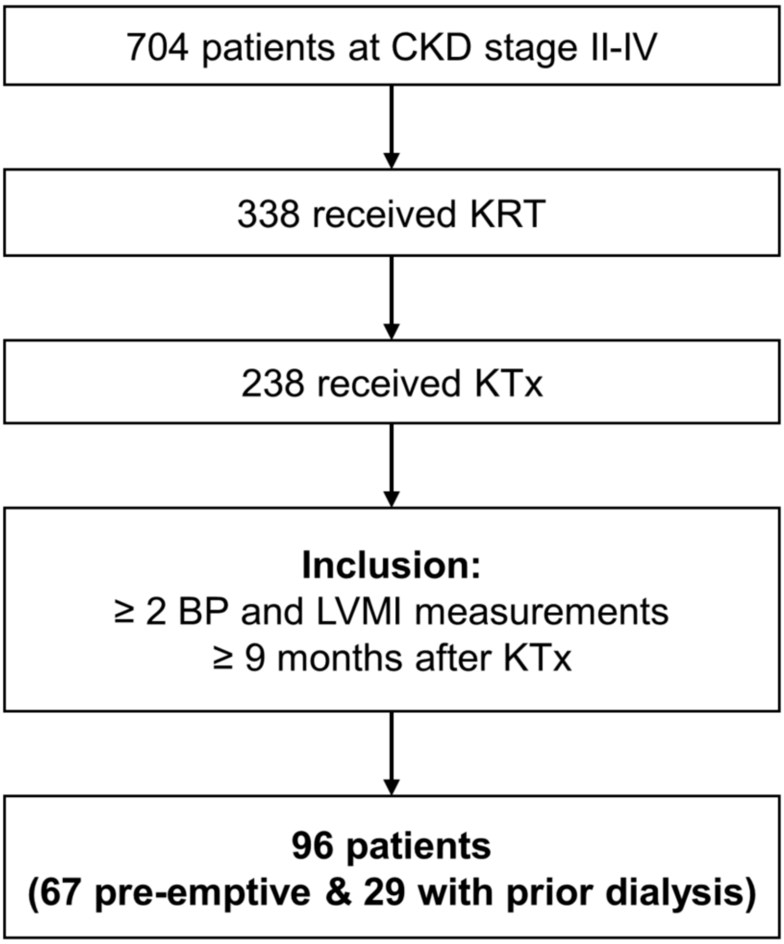

<u>Abbreviations:</u> BP, blood pressure; CKD, chronic kidney disease; KRT, kidney replacement therapy; KTx, kidney transplantation; LVMI, left ventricular mass index.

Supplemental Figure S2. Calculation of the cumulative BP exposure at each LVMI visit (vn) after baseline visit (v0).

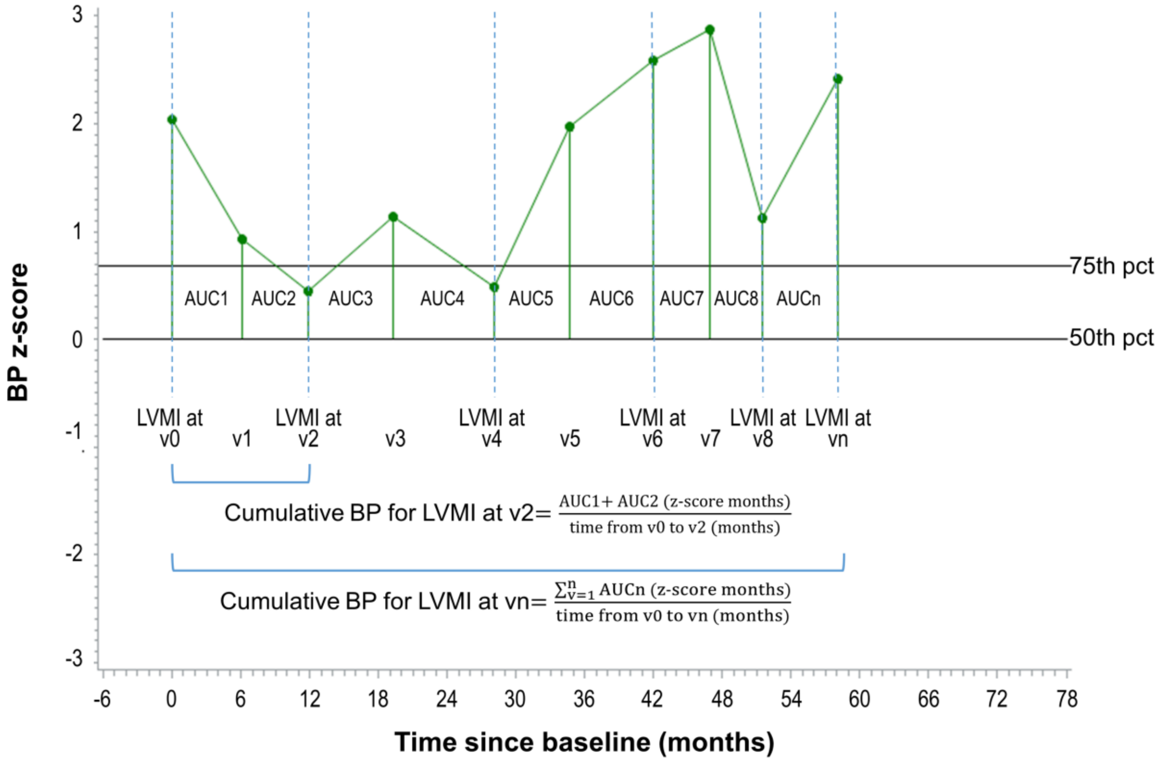

The area under the curve (AUC) of systolic and diastolic BP z-scores was calculated cumulatively for each visit with an LVMI measurement after the baseline visit (v0, first visit at ≥9 months after KTx). BP was measured every 6 months and LVMI yearly resulting in two BP measurements, which were obtained prior to and at the respective follow-up visit (a visit after baseline visit, vn), at which also an LVMI measurement took place. The cumulative BP at each LVMI follow-up visit was calculated using the AUC from baseline (v0) to each follow-up visit after the baseline visit (vn) averaged by time since v0 to vn (in months). The formula is given in the figure.

Abbreviations: *AUC, area under the curve; BP, blood pressure; KTx, kidney transplantation; LVMI, left ventricular mass index; pct, percentile; v, visit.*

Supplemental Figure S3. Unadjusted monotone spline regression fit showing the effect of cumulative SBP and DBP exposure on LVMI.

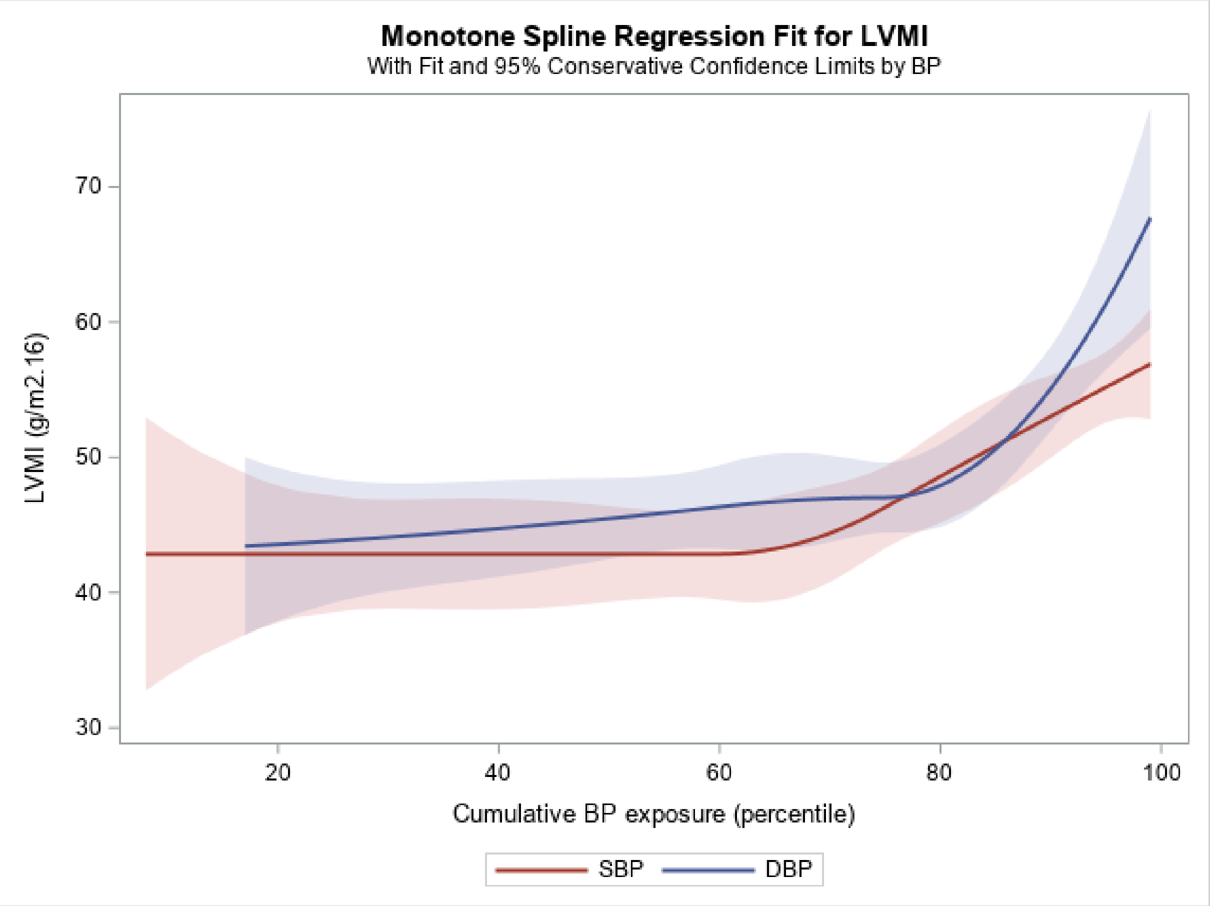

The spline regression of uncorrected data showed that LVMI increased above the 60th pct for SBP. With regard to DBP LVMI increased only slightly until the 80th pct and showed a steep increase thereafter.

<u>Abbreviations:</u> BP, blood pressure; DBP, diastolic blood pressure; LVMI, left ventricular mass index; SBP, systolic blood pressure.
